# Supplementary material for: Management Options for Fetal Bronchopulmonary Sequestration
Source: J Clin Med. 2022 Mar 20;11(6):1724. doi: 10.3390/jcm11061724 (PMC8954010; doi:10.3390/jcm11061724)
Supplement: Supplementary file 1 [file jcm-11-01724-s001.zip › jcm-1598977-supplementary.pdf]

Table S1. Data of fetuses with BPS managed expectantly.

Author	n	GA at diagnosis	Hydrops	Fetal / Neonatal death	Survival	GA at birth	Surgery
Langer *et al*., 1995 [32]	2	25 - 26	-	-	2 (100%)	37 - 38	0/lost to follow-up
Abuhamad *et al*., 1996 [33]	2	18	-	-	2 (100%)	39 – 40	0
da Silva *et al*., 1996 [34]	3	25 - 34	+ (2) ascites (1)	-	3 (100%)	30 - 35	3 (100%)
Evans *et al.,* 1996 [35]	3	25 - 34	+ (3)	-	3 (100%)	30 - 36	3 (100%)
Adzick *et al*., 1998 [1]	37	18 - 36	+ (1) ascites (1)	1 (neonatal)	36	no data	7 (19%)
Becmeur *et al*., 1998 [10]	8	20 - 33	-	-	8 (100%)	37 - 40	8 (100%)
Bratu *et al*., 2001 [36]	13	24	+ (2)	2 (neonatal)	11 (84%)	no data	11 (85%)
Chen *et al*., 2003 [37-39]	2	19 - 20	-	-	2 (100%)	38 - 40	1 (50%)
Illanes *et al*., 2005 [440]	4	19 - 29	-	-	4 (100%)	no data	2 (50%)
Ruano *et al*., 2005 [41]	3	21 - 33	-	1 (neonatal)	2 (77%)	no data	3 (100%)
Cavoretto *et al*., 2008 [22]	6	19 - 35	-	-	6 (100%)	37 - 42	5 (83%)
Mallmann *et al*., 2014 [17]	29	20 - 27	-	-	29 (100%)	34 - 39	16 (55%)
Stoiber *et al*, 2015 [18]	7	22	-		7 (100%)	39	no data
Nunes et al., 2015 [42]	7	[2nd – 3rd]			7 (100%)	>35	4 (57%)
Pinto *et al*., 2016 [43]	2	20 - 22	-		2 (100%)	34 - 38	0
Riley et al., 2018 [19]	100	19 - 37	5		100 (100%)	28 - 41	76 (76%)
Present series	16	20 - 28	-		16 (100%)	37 – 40	4 (25%)
Total	244		15/244 [6.1%]	4/244 [1.6%]	240/244 [98.3%]		143/226 [63.3%]

n=number of fetuses included in the study; GA = gestational age in weeks; Surgery = number of fetuses requiring postnatal surgery

Table S2a. Invasive management: data of fetuses with BPS treated with thoraco-amniotic shunt.

Author	n	GA at interv.	Method of treatment	Presence of hydrothorax /hydrops	Effect	Fetal death	Neonatal death	Survival	GA at birth	Surgery
Weiner *et al.*, 1986 [7]	1	24	Thoracocentesis + Thoraco-amniotic shunt	1 (100%)	Reaccumulated (100%)		1	0	29	1 (100%)
Slotnick *et al.*, 1990 [8]	1	32	Thoracocentesis + Thoraco-amniotic shunt	1 (100%)	Resolved (100%)			1 (100%)	34	1 (100%)
Hernanz-Schultzman *et al.*, 1991 [2]	1	27	Thoraco-amniotic shunt	1 (100%)	Resolved (100%)			1 (100%)	36	1 (100%)
Favre *et al.*, 1994 [9]	1	30	Thoraco-amniotic shunt	1 (100%)	Resolved (100%)			1 (100%)	38	1 (100%)
Adzick *et al.*, 1998 [1]	2	29-30	Thoraco-amniotic shunt	2 (100%)	Resolved (100%)			2 (100%)	33 - 35	2 (100%)
Becmeur *et al.,*1998 [10]	1	30	Thoraco-amniotic shunt	1 (100%)	Resolved (100%)			1 (100%)	38	1 (100%)
Lopoo *et al.*, 1999 [11]	2	23-30	Thoraco-amniotic shunt	2 (100%)	Resolved (100%)			2 (100%)	33	No data
Salomon *et al.*, 2003 [12]	1	34	Thoraco-amniotic shunt	1 (100%)	Resolved (100%)			1 (100%)	36	0
Picone et al., 2004 [13]	1	19-36	Thoraco-amniotic shunt	1 (100%)	No data (100%)			1 (100%)	28-40	No data
Hayashi *et al.*, 2006 [14]	3	28-30	Thoracocentesis + Thoraco-amniotic shunt	3 (100%)	Resolved (100%)			3 (100%)	33-35	3 (100%)
Odaka *et al.*, 2006 [15]	1	27	Thoraco-amniotic shunt	1 (100%)	Reaccumulated (100%)			1 (100%)	37	1 (100%)
Kitano *et al.*, 2006 [16]	3	27-30	Thoracocentesis + Thoraco-amniotic shunt	3 (100%)	Resolved (100%)			3 (100%)	33-35	No data
Mallmann *et al.*, 2014 [17]	7	25 - 29	Thoraco-amniotic shunt	7 (100%)	-	1		6 (85.7%)	30-37	5 (83%)
Stoiber *et al.*, 2015 [18]	2	29-30	Thoracocentesis + Thoraco-amniotic shunt	2 (100%)	Resolved (100%)		1	1 (50%)	33-36	0

Riley et al., 2018 [19]	2	23-27	Thoraco-amniotic shunt	2 (100%)	Resolved (100%)			2 (100%)	33-35	1 (50%)
Present series	5	20-25	Thoraco-amniotic shunt	5 (100%)	Resolved (100%)			5 (100%)	34-38	3 (60%)
	34			34/34 [100%]				31/34 [91.2%]		21/26 [84%]

n = number of fetuses included in the study; GA = gestational age in weeks; LB=life birth; IUD=intrauterine death; NND= neonatal death; Surgery = number of fetuses requiring postnatal surgery

Table S2b. Invasive management: data of fetuses with BPS treated with laser coagulation of the feeding vessel.

Author	No of fetuses	GA at interv.	Method of treatment	Presence of hydrothorax /hydrops	Effect	Fetal death	Neonatal death	Survival	GA at birth	Surgery
Oepkes *et al.*, (2007) [20]	1	23	Laser coagulation	1 (100%)	Resolved (100%)			1 (100%)	39	0
Ruano et al., 2007 [21]	1	29	Laser coagulation	1 (100%)	Resolved (100%)			1 (100%)	38	1 (100%)
Cavoretto et al., 2008 [22]	8	23-32	Laser coagulation	8 (100%)	Resolved (100%)			8 (100%)	34-41	5 (63%)
Witlox *et al.*, (2009) [23]	1	23	Laser coagulation	1 (100%)	Resolved (100%)			1 (100%)	41	0
Ruano et al., 2012 [24]	3	24-29	Laser coagulation	3 (100%)	Resolved (66.7%) No change (33.3%)	1		2 (66.7%)	30-39	No data
Mallmann *et al.*, 2014 [17]	5	24 - 31	Laser coagulation	5 (100%)	Resolved (100%)			5 (100%)	38 - 40	1 (20%)
Cruz-Martinez *et al.*, 2015, 2017 [25, 26]	15	20-31	Laser coagulation	15 (100%)	Resolved (100%)			15 (100%)	35-40	0
Kosinski *et al.*, 2017 [27]	2	22-27	Laser coagulation	2 (100%)	Resolved (100%)			2 (100%)	39-41	0
Gottschalk *et al.*, 2017 [28]	11	25-34	Laser coagulation	11 (100%)	Resolved (100%)			11 (100%)	30-42	3 (27%)

Present series	7	21-25	Laser coagulation	7 (100%)	Resolved (100%)			7 (100%)	39-41	2 (28.6%)
Total	54							53/54 [98.1%]		12/51 [23.5%]

N = number of fetuses included in the study; GA = gestational age in weeks; LB=life birth; IUD=intrauterine death; Surgery = number of fetuses requiring postnatal surgery

Table S2c. Invasive management: data of fetuses with BPS treated with intratumor injection of sclerosant or combined treatment.

**Author**	**No of fetuses**	**GA at interv.**	**Method of treatment**	**Presence of hydrothorax /hydrops**	**Effect**	**Fetal death**	**Neonatal death**	**Survival**	**GA at birth**	**Surgery**
Nicolini et al. 2000 [29]	1	27	Alcohol injection + thoracoamniotic shunt	1 (100%)	Resolved (100%)			1 (100%)	40	0
Bermudez *et al*., 2007 [30]	3	24-26	Polidocanol injection	2 (67%)	Resolved (100%)		1	2 (67%)	38	2 (67%)
Rammos KS *et al.*, 2010 [31]	2	30-31	Thoracoamniotic shunt + laser coagulation + thoracocentesis	1 (100%)	Resolved (100%)			2 (100%)	No data	2 (100%)
Gottschalk *et al.*, 2017 [28]	1	25, 26	Thoracoamniotic shunt + laser coagulation	1 (100%)	Resolved (100%)			1 (100%)	40	0
Present series	1	25	Thoracoamniotic shunt + laser coagulation	1 (100%)	Resolved (100%)			1 (100%)	40	1 (100%)
Total	8							7/8 [87.5%]		5/8 [62.5%]

N = number of fetuses included in the study; GA = gestational age in weeks; LB=life birth; NND= neonatal death; Surgery = number of fetuses requiring postnatal surgery
